# Glabridin Prevents Doxorubicin-Induced Cardiotoxicity Through Gut Microbiota Modulation and Colonic Macrophage Polarization in Mice

**DOI:** 10.3389/fphar.2019.00107

**Published:** 2019-02-15

**Authors:** Keqing Huang, Yanzhuo Liu, Honglin Tang, Miao Qiu, Chenhong Li, Chenfan Duan, Chenlong Wang, Jing Yang, Xiaoyang Zhou

**Affiliations:** ^1^Department of Cardiology, Renmin Hospital, Wuhan University, Wuhan, China; ^2^Hubei Province Key Laboratory of Allergy and Immune-Related Diseases, Department of Pharmacology, School of Basic Medical Sciences, Wuhan University, Wuhan, China; ^3^Hubei Key Laboratory of Medical Information Analysis & Tumor Diagnosis and Treatment, Key Laboratory of Cognitive Science, College of Biomedical Engineering, South Central University for Nationalities, Wuhan, China; ^4^Shenzhen Stomatological Hospital of Southern Medical University, Shenzhen, China; ^5^Laboratory of Membrane Ion Channels and Medicine, Key Laboratory of Cognitive Science, State Ethnic Affairs Commission, College of Biomedical Engineering, South Central University for Nationalities, Wuhan, China

**Keywords:** glabridin, cardiotoxicity, doxorubicin, gut microbiota, colonic macrophage

## Abstract

The chemotherapeutic drug doxorubicin (DOX) provokes a dose-related cardiotoxicity. Thus, there is an urgent need to identify the underlying mechanisms and develop strategies to overcome them. Here we demonstrated that glabridin (GLA), an isoflavone from licorice root, prevents DOX-induced cardiotoxicity through gut microbiota modulation and colonic macrophage polarization in mice. GLA reduced DOX-induced leakage of myocardial enzymes including aminotransferase, creatine kinase, lactate dehydrogenase, and creatine kinase-MB. GLA downregulated pro-apoptotic proteins (Bax, cleaved-caspase 9 and cleaved-caspase 3) and upregulated anti-apoptotic proteins (HAX-1 and Bcl-2) in the cardiac tissues. In addition, GLA modulated DOX-induced dysbiosis of gut microbiota and thereby decreased the ratio of M1/M2 colonic macrophage, accompanied by the downregulated lipopolysaccharide (LPS) and upregulated butyrate in the feces and peripheral blood. The leakage of myocardial enzymes induced by the DOX was decreased by antibiotics treatment, but not altered by co-treatment with the GLA and antibiotics. The ratio of M1/M2 colonic macrophage and leakage of myocardial enzymes reduced by the GLA were greatly increased by the *Desulfovibrio vulgaris* or LPS but decreased by the butyrate. Depletion of the macrophage attenuated DOX-induced cardiotoxicity but failed to further affect the effects of GLA. Importantly, GLA decreased production of M1 cytokines (IL-1β and TNF-α) but increased production of M2 cytokines (IL-10 and TGF-β) in the colonic macrophage with the downregulation of NF-κB and the upregulation of STAT6. In summary, GLA prevents DOX-induced cardiotoxicity through gut microbiota modulation and colonic macrophage polarization, and may serve as a potential therapeutic strategy for the DOX-induced cardiotoxicity.

## Introduction

The anthracycline doxorubicin (DOX) is one of the most effective chemotherapeutic agents used for cancer treatment. However, its clinical use is limited by dose-related cardiotoxicity caused by various mechanisms including oxidative stress (Baxter-Holland and Dass, 2018). Most of the mechanisms ultimately result in the activation of apoptotic signaling, leading to progressive loss of cardiac myocytes (Octavia et al., 2012). Considering the limited capacity for the cardiac cell proliferation, it is essential to effectively prevent cardiac cell apoptosis, and thereby overcome DOX-induced cardiotoxicity.

Intestinal microbiota plays an essential role in host physiology (van Vliet et al., 2010). Intestinal microbiota has also been shown to promote cardiovascular disease, including atherosclerosis and heart failure (Yamashita et al., 2015). Lipopolysaccharide (LPS), one of cell wall components from Gram-negative bacteria, promotes production of inflammatory cytokines in cardiovascular disease (Zöllner et al., 2017). LPS and its receptor, Toll-like receptor 4 (TLR4) drive DOX-induced damages in heart, kidney, liver, and intestine (Wang et al., 2016). Conversely, gut microbiota exerts a beneficial effect on host health through production of short-chain fatty acids (SCFAs). Importantly, butyrate has received most attention out of the SCFAs as a key mediator of anti-inflammation (Brahe et al., 2013). Targeting the gut microbiota and their products prevents atherosclerotic cardiovascular diseases, and may be a potential therapeutic strategy (Jie et al., 2017).

Gut microbiota dysbiosis promotes M1 polarization of the macrophage in adipose tissue in obesity (Hersoug et al., 2016). Functionally differentiated macrophages are commonly subdivided into two categories, the classically activated M1-like phenotype, which produces pro-inflammatory factors, and the alternatively activated M2-like phenotype, which produces anti-inflammatory factors (Hu et al., 2015). DOX administration significantly promotes production of the pro-inflammatory factors IL-1β and TNF-α by the M1-like macrophage, while a decrease in their production alleviates DOX-induced cardiac damage (Kobayashi et al., 2016; Johnson and Singla, 2018). Previous study showed that the release of pro-inflammation cytokines from LPS-induced mouse peritoneal macrophages promotes DOX-induced heart damage (Wang et al., 2016). Conversely, the M2-like macrophage, characterized by production of IL-10 and TGF-β, exerts cardioprotective effects in heart failure (Fujiu et al., 2014; Hulsmans et al., 2016). The colonic macrophage is one of the most abundant leukocytes in the intestines of all mammals (Kawano et al., 2016). In the steady state, the colonic macrophage exhibits an anti-inflammatory M2-like phenotype. The pro-inflammatory M1-like macrophage has been detected in the colon from animal models of inflammatory bowel disease (IBD) and patients with IBD (Isidro and Appleyard, 2016). Inhibition of pro-inflammatory responses in the colonic macrophage prevents dextran sulfate sodium (DSS)-induced intestinal injury and colitis (Huang et al., 2015). However, whether colonic macrophages and their polarization contribute to DOX-induced cardiotoxicity have not been explored.

Accumulating evidence suggests that flavonoids including quercetin, baicalei, and catechin exert significant protective effects on DOX-mediated oxidative stress and cardiotoxicity (Shabalala et al., 2017). Quercetin and catechin, two bioactive flavonoids in plants, modulate the composition of the gut microbiota evidenced by potentiating the growth of specific beneficial bacteria strains including *Lactobacillus* and *Bifidobacterium* and inhibiting the growth of certain pathogenic bacteria including *Clostridium* (Etxeberria et al., 2013; Pérez-Cano et al., 2014). Licorice has been used for detoxification and treatment for injury in China as described in first Chinese dispensatory (Nomura and Fukai, 1998). The hexane/ethanol extract from *Glycyrrhiza uralensis* suppresses DOX-induced apoptosis *in vivo* and *in vitro* (Choi et al., 2008; Zhang et al., 2011). GLA, an isoflavone derived from licorice root, exhibits various biological properties, such as anti-inflammation, anti-bacterium and anti-oxidation (Simmler et al., 2013). GLA also prevents low density lipoprotein (LDL) oxidation-induced atherogenic processes and cardiovascular injury (Simmler et al., 2013). However, whether GLA exerts protective effects against DOX-induced cardiotoxicity is not known. Here we demonstrated that GLA indeed protects against DOX-induced cardiotoxicity in mice through prevention of gut microbiota dysbiosis and alteration of colonic macrophage phenotype. Our findings demonstrate pharmacologic use of GLA in protecting against DOX-induced cardiotoxicity through a novel link between the gut microbiota, colonic macrophage polarization and cardiotoxicity.

## Materials and Methods

### Chemicals and Reagents

Glabridin (53633) with high purity (≥98%) was purchased from Sigma Chemical Co. (St. Louis, MO, United States). GLA was dissolved in 0.9% sodium chloride solution containing 1% (w/v) sodium carboxymethylcellulose (CMC-Na). Doxorubicin hydrochloride injection (1703E4, Adriamycin 10 mg) was purchased from Shenzhen Main Luck (Shenzhen, China). Clodronate (69008214) was purchased from Sinopharm Chemical Reagent (Shanghai, China). LPS (L2630) and sodium butyrate (ARK2161) were purchased from Sigma-Aldrich (St. Louis, MO, United States). The antibodies against β-actin (ab8226), cleaved capase 3 (ab13847), cleaved capase 9 (ab202068), toll-like receptor 4 (TLR4, ab13556), IκBα (ab32518) and CD68 (ab125212) were purchased from Abcam (Cambridge, MA, United States). The antibodies against PerCP-Cy5.5-conjugated anti-CD11c (560584) and PE-conjugated anti- CD11b (561689) were purchased from BD Biosciences (Oxford, United Kingdom). Antibody against FITC-conjugated anti- F4/80 (130-117-509) was purchased from Miltenyi Biotec (Bergisch Gladbach, Germany). The antibodies against induced nitric oxide synthase (iNOS, sc-651) and CD206 (sc-48758) were purchased from Santa Cruz Biotechnology (Santa Cruz, CA, United States). Antibodies against B-cell lymphoma protein 2-associated X (Bax, 2772), B-cell lymphoma-2 (Bcl-2, #3498), phosphor- IκBα (#2859), signal transducers and activators of transcription (STAT) 6 (#5397), p-STAT6 (#56554), NF-κB p65 (#8242) and phosphor-NF-κB p65 (#3033) were purchased from Cell Signaling Technology (Danvers, MA, United States). Antibodies against HS-associated rotein X-1 (HAX-1, ABT65) was purchased from Merck Millipore (Billerica, MA, United States).

### Preparation of the Clodronate Liposome

The preparation of the clodronate liposome was based on previous descriptions (Van Rooijen and Sanders, 1994). Briefly, 8 mg of cholesterol and 86 mg of phosphatidylcholine (69014933, Sinopharm Chemical Reagent Co., Ltd., Shanghai, China) were combined with 10 ml of a clodronate (69008214, Sinopharm Chemical Reagent Co., Ltd., Shanghai, China) solution and sonicated gently. The resulting liposome was then washed to eliminate free drug. For control experiment, phosphate buffer saline (PBS)-filled liposome was prepared under the same conditions.

### Animals and Treatments

C57BL/6 mice (male, 6–8 weeks old) were purchased from Centers for Disease Control and Prevention (Hubei, China). The mice were acclimatized 1 week to adapt to the new environment before the experiment. All mice were fed standard chow diet and tap water *ad libitum*, and housed at room temperature (18–22°C) under a 12-h light/12-h dark cycle. All mice experiments were approved by the Animal Research Committee of Wuhan University and maintained in accordance with the guidelines by the Association for Assessment and Accreditation of Laboratory Animal Care International (AAALAC). The mice were fed purified diets (D12450B; Research Diets Inc., New Brunswick, NJ, United States), and were caged individually to avoid cage effects.

A mice model of DOX-induced cardiotoxicity was established as previously described (Cao et al., 2014). Briefly, mice were randomly divided into four groups (*n* = 10 per group): DOX, DOX plus GLA (15 and 30 mg/kg) and control groups. Mice in the DOX group were intraperitoneally administered with a single dose of DOX (20 mg/kg). Mice in the DOX plus GLA (15 and 30 mg/kg) groups were treated with GLA at the dose of 15 and 30 mg/kg once daily *via* oral gavage for 12 days, starting 7 days before DOX injection. The doses of GLA used in the present study were based on the published study (Kwon et al., 2008) and our preliminary experiments. Mice in the control group were received an equivalent volume of 1% CMC-Na *via* oral gavage and saline by intraperitoneal injection. The food intake was measured per animal. The mice were euthanized 5 days after the injection of DOX.

For antibiotic treatment, mice from the broad-spectrum antibiotics (Abx) and GLA plus Abx groups were treated with a mixture of 0.5 g/L vancomycin, 0.5 g/L neomycin sulfate and 0.5 g/L primaxin in the drinking water for 2 weeks, which was refreshed every 2 days (Iida et al., 2013). After antibiotic treatment, mice were treated with GLA at the dose of 30 mg/kg once daily *via* oral gavage for 12 days, starting 7 days before DOX injection. The mice were euthanized 5 days after the injection of DOX.

For exogenous supplement experiment of the microbial products (LPS and butyrate), the mice from DOX plus GLA (30 mg/kg) groups as above described were randomly divided into four groups (*n* = 10 per group): *Desulfovibrio vulgaris* (Des), LPS, butyrate and vehicle groups. The GLA treatment was continued throughout the experiment period among the groups. The mice were injected intraperitoneally with LPS (1 mg/kg), and orally administrated with sodium butyrate (1 g/kg/day) or *D. vulgaris* (ATCC 29579, 1× 10^9^ CFU/mouse/every 2 days) 1 h after the injection of DOX. The dose of sodium butyrate and LPS used in the present study were based on the published studies (Chen X. et al., 2017; Li et al., 2018) and our preliminary experiments. The mice were euthanized 5 days after the injection of DOX.

To confirm that any beneficial effects observed reflect macrophage depletion rather than off-target effects of the drug, we used the clodronate liposome (Clod) to deplete the macrophage as our previous research (Chen X.W. et al., 2017). Clod was administered beginning 1 h after DOX injection to deplete the macrophage. Mice were injected intraperitoneally with Clod (100 mg/kg), followed by repeated injections of 50 mg/kg every 4th day to prevent repopulation of the macrophage. The Clod doses were based on the literature, and the efficiency of macrophage depletion was assessed by immunostaining for F4/80, as previously described (Zhang et al., 2010). The mice were euthanized 5 days after the injection of DOX.

### Fecal Microbiota Analysis by 16S rRNA Sequencing

Fresh stool pellets from the DOX, DOX plus GLA (30 mg/kg) and control groups were obtained before mice were euthanized, and immediately stored at -80°C. The fecal microbiota analysis by 16S rRNA sequencing was based on previous description (Sampson et al., 2016). Bacterial DNA was extracted using TIANamp stool DNA kit (DP328-02, TIANGEN Biotech CO., Ltd., Beijing, China). DNA concentration and integrity were determined both visually by electrophoresis on a 1% agarose gel containing ethidium bromide and spectrophotometrically by using a Nanodrop instrument (Thermo Scientific). The V3–V4 region of the 16S rRNA gene was amplified using a set of primers (338F: 5′-GTGCCAGCMGCCGCGGTAA-3′ and 806R: 5′-GGACTACHVGGGTWTCTAAT-3′). Sequencing was performed by an Illumina MiSeq PE300 system (OE Biotech Co., Ltd.). Paired-end sequences were merged to give an optimal alignment (overlap length ≥ 10 bp, mismatch proportion ≤ 20%). As an added quality control measure, the software package MacQIIME (version 1.9.1) pipeline was used to filter out and discard poor-quality reads using the default settings. Sequences were further clustered into OTUs (Operational Taxonomic Units or phylotypes) at 97% of identity using QIIME and cdhit. OTUs were assigned to closest taxonomic neighbors and relative bacterial species using Seqmatch and Blastall. Relative abundance of each OTUs and other taxonomic levels (from phylum to genus) was calculated for each sample to account for different levels of sampling across multiple individuals. Principle Coordinate Analysis (PCoA) projections were visualized using Emperor 0.9.4. The obtained sequences were deposited in a NCBI Sequence Read Archive under accession number RPJNA508594.

### Analysis for SCFA in the Feces and Peripheral Blood

The levels of SCFAs in the feces and peripheral blood from the DOX, DOX plus GLA (30 mg/kg) and control groups were analyzed by high performance liquid chromatography (HPLC) based on previous description (Sampson et al., 2016). Feces (100 mg) were mashed in 500 μl HPLC grade water and centrifuged at 14,000 *g*, the resulting supernatants were then passed through a 0.22 μm syringe filter to remove bacterial cells and debris. The peripheral blood was obtained *via* the retro-orbital vein blood of individual mice and put at room temperature for 30 min. Serum was carefully separated by centrifuged for 10 min at 3,000 *g* from peripheral blood. The supernatants or serum were then acidified with 1/10 volume of 0.01 M H_2_SO_4_, heated, and passed through a condenser to isolate volatile compounds. SCFAs analyses were performed using an Agilent 1200 series HPLC and a Poroshell 120 SB C18 column (2.7 μm, 3.0 × 100 mm) with guard column (Agilent Technologies). 0.01 M H_2_SO_4_ was used as the mobile phase. SCFAs was identified by comparing sample peak retention times to a standard volatile acid mix (Matreya 1075) and concentrations were determined by UV monitor (Bio-Rad Model 1801) at a wavelength of 220 nm.

### LPS Quantification in the Feces and Peripheral Blood

Lipopolysaccharide quantification in the feces and peripheral blood from the DOX, DOX plus GLA (30 mg/kg) and control groups was performed using the Chromo-Limulus Amebocyte Lysate (Chromo-LAL) reagent (#C0031-5, Associates of Cape Cod, Inc.) as described previously (Lau et al., 2016). Briefly, 1 mL of sterile saline solution (NaCl 0.9%) was added to 100 mg feces, vortexed and centrifuged (10 min, 10,000 *g*, 4°C) twice. The supernatant was then filtered with 0.22 μm filter. The peripheral blood was obtained from mice and put at room temperature for 30 min. Serum was carefully separated by centrifuged for 10 min at 3,000 *g* from peripheral blood *via* the retro-orbital vein blood. The supernatants or serum were mixed with Chromo-LAL (1:1) and incubated at 37°C for 20 min. Absorbance was read every 10 s at 405 nm.

### Isolation and Culture of the Colonic Macrophages

The colonic macrophages were isolated by flow cytometry as described previously (Kawano et al., 2016). Briefly, the colons were removed from all intestinal fats and the cecum, cut open, washed in PBS (Ca^2+^ and Mg^2+^ free) before being dissected and cut into 0.5 cm pieces. The tissues were then incubated with 0.25% tyrisin at 37°C for 30 min. Supernatants were harvested and passed through a 100 μm strainer, and stained with PerCP-Cy5.5-conjugated anti-CD11c antibody, FITC-conjugated anti-F4/80 antibody and PE-conjugated anti-CD11b antibody. The stained cells were then sorted using a FACSAria Cell Sorter. The purity of the colonic macrophage was >95%. Freshly isolated colonic macrophages from each treatment group were incubated in 12-well plates at 80% to 90% confluence, washed with PBS, and resupplied with serum-free RPMI 1640 media for 24 h. The supernatants and colonic macrophages were collected, respectively, for further experiments.

### TUNEL Assay

The apoptotic cell death was evaluated by the TUNEL (Terminal deoxynucleotidyl transferase dUTP nick end labeling) method using an apoptosis detection kit (C1090, Beyotime Biotechnology, Shanghai, China). The cardiac tissues from the DOX, DOX plus Clod, DOX plus GLA and DOX plus GLA plus Clod groups were embedded in paraffin. The sections were cut to 4 μm thicknesses and mounted on polylysine coated glass slides. Immunofluorescence procedures for detecting apoptotic cardiomyocytes were performed according to the manufacturer’s instructions by using an Olympus BX51 microscope (Olympus, Tokyo, Japan). For each slide, 10 fields were randomly chosen, and using a defined rectangular field area (40× objective). The index of apoptosis was determined (number of positively stained apoptotic myocytes/rectangular field area) from a total of 100 fields per heart. Assays were performed in a blinded manner.

### Detection of Serum Biochemical Indexes

The serum levels of aminotransferase (AST) (C010-2), creatine kinase (CK) (A032), lactate dehydrogenase (LDH) (A020-2) and creatine kinase-MB (CK-MB) (E006) were detected according to the instructions of kits (Nanjing Jiancheng Bioengineering Institute, Nanjing, China).

### Immunofluorescence

The colonic tissue sections from the DOX, DOX plus GLA, LPS, butyrate and *D. vulgaris* groups were labeled with anti-CD68 (1:100), anti-CD206 (1:100), and anti-iNOS (1:100) antibodies overnight at 4°C. After washing, the sections were incubated with a mixture of Alexa Fluor 488- and Alexa Fluor 594-conjugated secondary antibodies for 1 h. Nuclei were counterstained with 4′, 6-diamidino-2-phenylindole (DAPI). Each section was observed by using an Olympus BX51 microscope (Olympus, Tokyo, Japan). For quantification, the index of positively stained cells was determined (number of CD68^+^ iNOS^+^ and CD68^+^ CD206^+^ cells/rectangular field area) from 10 randomly chosen fields at a magnification of 400× for each sample.

### Histopathological Examination

The cardiac tissue sections from the DOX, DOX plus GLA (15 and 30 mg/kg) or control groups were stained with Hematoxylin and Eosin (H&E) or Masson’s Trichrome. The slides were evaluated by two pathologists who were masked to the treatment protocol and results. For quantification, Masson’s Trichrome staining for interstitial fibrosis has been done using ImageJ software (1.41v, US National Institutes of Health, United States), and the percentage of the area of fibrosis to that of the entire tissue specimen was calculated. The number of vacuolated cardiomyocytes was counted in 10 randomly chosen fields at a magnification of 200× for each sample.

### Western Blot Analysis

Protein in the cardiac tissues and colon tissues from the DOX, DOX plus GLA (15 and 30 mg/kg) or control groups were extracted. Based on the concentration determined by bicinchoninic acid (BCA) method, equal amounts of protein extracts were subjected to electrophoresis in SDS-polyacrylamide gels and transferred to polyvinylidene fluoride (PVDF) membranes. The membrane was blocked with 5% non-fat milk and probed with primary antibodies against CD68 (1:2000), TLR4 (1:2000), cleaved caspase 9 (1:1000), cleaved caspase 3 (1:1000), iNOS (1:200), CD206 (1:200), Bax (1:1000), Bcl-2 (1:1000) β-actin (1:1000), STAT6 (1:1000) and p-STAT6 (1:1000), NF-κB p65 (1:1000), phosphor-NF-κB p65 (1:1000), HAX-1 (1:200). The antibodies were detected using peroxidase-conjugated anti-rabbit and anti-mouse immunoglobulin G (CST), and blots were detected using the ECL system (Thermo Fisher Scientific, Waltham, MA, United States). Band intensity was quantified by densitometric analysis using the ImageJ software (1.41v, US National Institutes of Health, United States). The relative expression was normalized to the expression of β-actin.

### Cytokine Measurement

M1 cytokines TNF-α (SMTA00B) and IL-1β (SMLB00C) and M2 cytokines IL-10 (SM1000B) and TGF-β (SMB100B) in the colonic macrophages from the mice treated with the DOX, GLA (15 and 30 mg/kg) or vehicle were measured by ELISA (R&D Systems, Minneapolis, MN, United States).

### qPCR Analysis

Total RNA was extracted from isolated colonic macrophages with Trizol (Invitrogen, Carlsbad, CA, United States) according to the manufacturer’s instruction. RNA concentration and purity were estimated from the optical density at 260 and 280 nm, respectively. Total RNA was subjected to cDNA synthesis using M-MLV reverse transcriptase. PCR amplification was performed with the CFX96 Real Time System (Bio-Rad, Hercules, CA, United States), using SYBR Premix Ex Taq TM (2×; Takara, Japan). The primers of M1 markers (iNOS and CXCL9), M2 markers (arginase-1 and CD206) and *D. vulgaris* were designed according to previous studies (Figueiredo et al., 2013; Chen X.W. et al., 2017) and the sequence of each primer was queried using the National Center for Biotechnology Information BLAST database for homology comparison. Relative quantification was performed using the 2^-ΔΔ^*^C^*^t^ method. Fecal samples were normalized using *D. vulgaris* 16S rRNA. Relative expression levels of the M1 and M2 markers were normalized with the β-actin. The sequences of all primers were listed in Supplementary Table S1.

### Statistical Analysis

The values were expressed as mean ± SEM, and statistical analyses were performed using a one-way ANOVA followed by the Student–Newman–Keuls test. The data of microbiome abundance were analyzed by non-parametric Mann–Whitney *U* test correcting for multiple comparisons. *P*-values of 0.05 or less were considered significant.

## Results

### GLA Prevents DOX-Induced Cardiotoxicity in Mice

**FIGURE 1:**
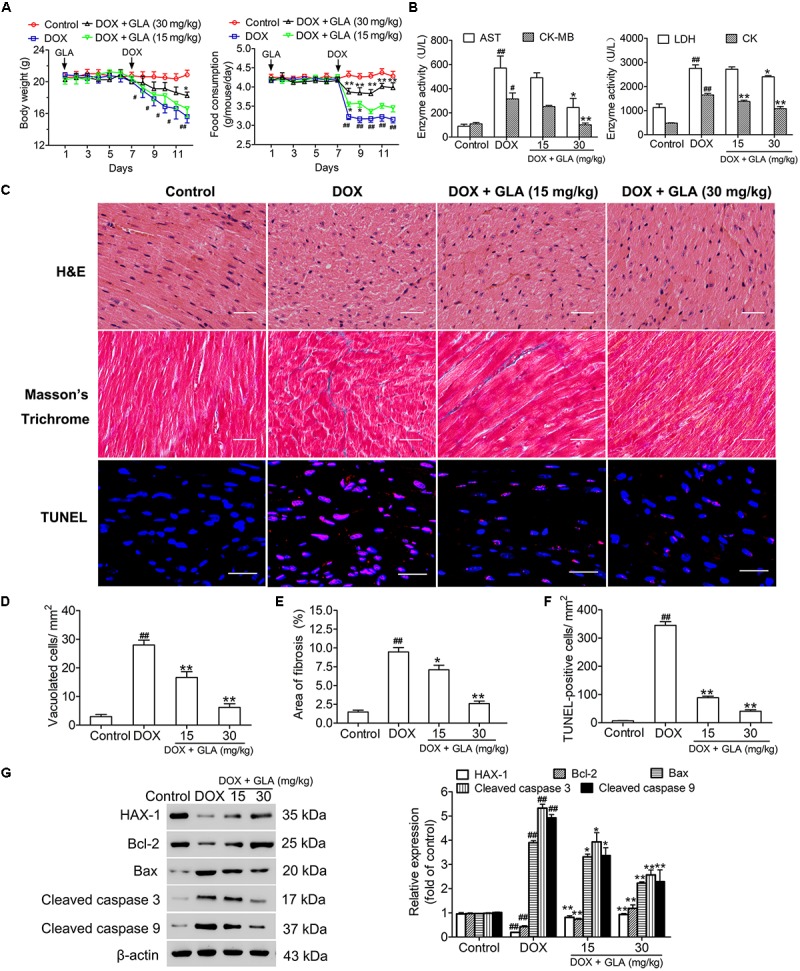
Glabridin (GLA) prevents doxorubicin (DOX)-induced cardiotoxicity in mice. A single dose of DOX (20 mg/kg) was intraperitoneally injected into the C57BL/6 mice to induce acute cardiotoxicity. GLA (15 and 30 mg/kg) or vehicle was intragastrically administered once daily for 12 days, starting 7 days before DOX injection. **(A)** Changes in body weights and food intake (*n* = 10). **(B)** Serum levels of AST, CK-MB, LDH, and CK (*n* = 10). **(C)** Histological assessment of hearts (*n* = 10). Hematoxylin and eosin (H&E) was performed to evaluate myocardial vacuolization. Scale bars, 50 μm. Masson staining was performed to evaluate myocardial fibrosis. Scale bars, 50 μm. TUNEL assay was performed to assess the cardiomyocytes apoptosis. Scale bars, 20 μm. **(D)** Quantitative result of vacuolated cells. **(E)** Quantitative result of myocardial fibrosis area (*n* = 10). **(F)** Quantitative result of TUNEL-positive cells (*n* = 10). **(G)** HAX-1, Bcl-2, Bax, cleaved caspase-3 and cleaved caspase-9 were determined by Western blot (*n* = 10). The values are presented as the mean ± SEM. ^#^*P* < 0.05, ^##^*P* < 0.01 vs. control, ^∗^*P* < 0.05, ^∗∗^*P* < 0.01 vs. DOX.

The body weights and food intake of all mice were monitored throughout the study. As shown in Figure 1A, the mice lost weight and food intake after administration of DOX. However, the GLA (15 and 30 mg/kg)-treated mice maintained body weight and food intake better than the DOX-treated mice. DOX significantly increased serum AST, CK-MB, LDH, and CK levels as compared to the control. Conversely, GLA (30 mg/kg) significantly decreased serum AST, CK-MB, LDH, and CK levels as compared to the DOX-treated mice (Figure 1B). Next, we evaluated histological alterations of cardiac tissues, and found that DOX led to serious disorganization of myofibrillar arrays, cytoplasmic vacuolization and intense infiltration of neutrophils in the cardiac tissues as compared to the control (Figure 1C,D). In contrast, GLA restored the cardiac architecture toward normal in a dose (15 and 30 mg/kg)-dependent manner (Figure 1C,D). Masson’s Trichrome confirmed the significant increase in the areas of interstitial fibrosis in the cardiac tissues from the DOX-treated mice, which was attenuated by treatment with GLA (15 and 30 mg/kg) (Figure 1C,E). TUNEL assay was performed to assess the cardiomyocyte apoptosis. As shown in Figure 1C,F, TUNEL-positive cardiomyocytes were more frequently observed in the DOX-treated mice as compared with the control, which was alleviated by GLA (15 and 30 mg/kg). Western blot analysis revealed that GLA suppressed DOX-induced cleaved-caspase 3 and cleaved-caspase 9 activation. While DOX suppressed expression of anti-apoptotic proteins HAX-1 and Bcl-2, GLA dramatically upregulated the proteins expression of HAX-1 and Bcl-2 (Figure 1G). These results suggest that GLA is effective in protecting against DOX-induced cardiotoxicity in mice.

### Modulation of Gut Microbiota Dysbiosis by GLA Prevents DOX-Induced Cardiotoxicity in Mice

**FIGURE 2:**
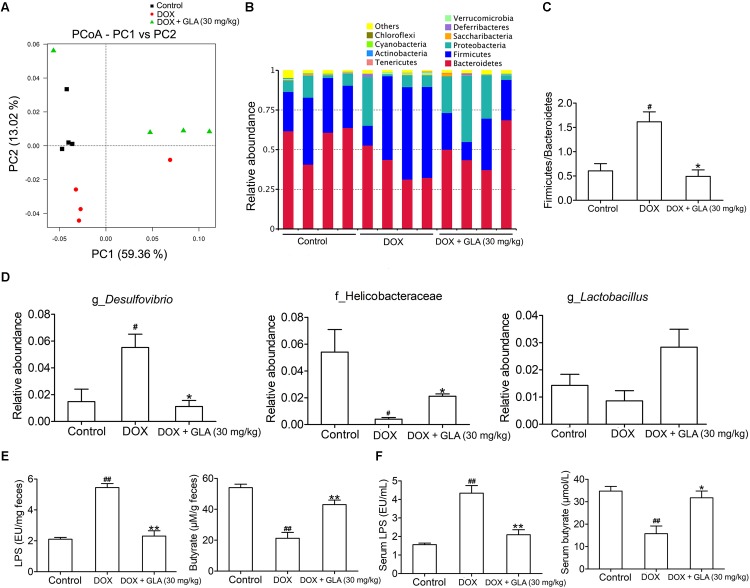
Glabridin (GLA) prevents doxorubicin (DOX)-induced dysbiosis of gut microbiota in mice. A single dose of DOX (20 mg/kg) was intraperitoneally injected into the C57BL/6 mice to induce acute cardiotoxicity. GLA (30 mg/kg) or vehicle was intragastrically administered once daily for 12 days, starting 7 days before DOX injection. **(A)** Principal co-ordinates analysis plot of bacterial β-diversity (*n* = 4). **(B)** The relative taxonomic abundance at the phylum level of gut microbiota (*n* = 4). **(C)** The ratios of Firmicutes/Bacteroidetes (*n* = 4). **(D)** The relative abundance of *Desulfovibrio* genus, helicobacteraceae family and *Lactobacillus* genus (*n* = 4). **(E,F)** Lipopolysaccharide (LPS) and butyrate levels in feces and peripheral blood were determined by Limulus Amebocyte Lysate (LAL) assays or high performance liquid chromatography (HPLC), respectively (*n* = 10). The values are presented as the mean ± SEM. ^#^*P* < 0.05, ^##^*P* < 0.01 vs. control, ^∗^*P* < 0.05, ^∗∗^*P* < 0.01 vs. DOX.

Among 12 chemotherapeutic agents commonly used to treat cancer, DOX induces great variation in gut microbiota (Rawls et al., 2006; Flórez et al., 2016). Thus, we sequenced the bacterial 16S rRNA in the feces of mice. UniFrac-based principal coordinates analysis revealed obvious difference in a distinct clustering of fecal microbial structure among the DOX, DOX plus GLA and control groups (Figure 2A). This was further supported by the obvious different abundance of bacterial phyla among the three groups (Figure 2B). We observed that DOX increased the ratio of Firmicutes/Bacteroidetes, a marker of gut dysbiosis (Shin et al., 2015). Conversely, GLA (30 mg/kg) decreased the ratio of Firmicutes/Bacteroidetes to the levels similar to that of the control mice (Figure 2C). Furthermore, GLA (30 mg/kg) significantly decreased the abundance of *Desulfovibrio* genus but increased the abundance of Helicobacteraceae family and *Lactobacillus* genus as compared with the DOX-treated mice (Figure 2D). Importantly, DOX increased the LPS levels and decreased butyrate levels in the feces and peripheral blood, which were attenuated by GLA (30 mg/kg) (Figure 2E,F).

**FIGURE 3:**
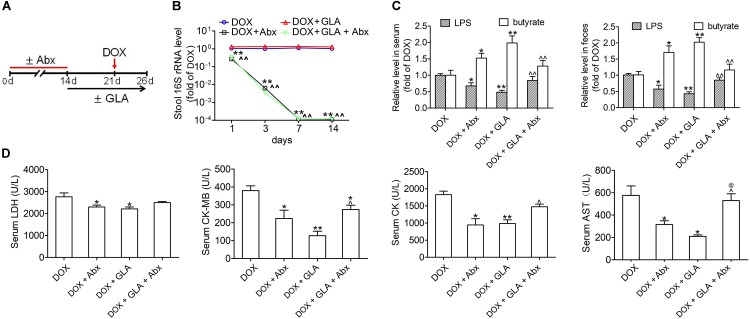
Glabridin (GLA) prevents doxorubicin (DOX)-mediated cardiotoxicity though modulation of gut microbiota. The C57BL/6 mice from broad-spectrum antibiotics (Abx) and GLA (30 mg/kg) plus Abx groups were treated with a mixture of 0.5 g/L vancomycin, 0.5 g/L neomycin sulfate and 0.5 g/L primaxin in the drinking water for 2 weeks. After antibiotic treatment, a single dose of DOX (20 mg/kg) was intraperitoneal injected into the C57BL/6 mice to induce acute cardiotoxicity. GLA (30 mg/kg) or vehicle was intragastrically administered once daily for 12 days, starting 7 days before DOX injection. **(A)** Schematic representation of the experimental approach. **(B)** The efficacy of Abx to remove gut bacteria was confirmed by quantifying stool bacterial load (*n* = 10). **(C)** The levels of Lipopolysaccharide (LPS) and butyrate levels in feces and peripheral blood were determined by Limulus Amebocyte Lysate (LAL) assays or high performance liquid chromatography (HPLC), respectively (*n* = 10). **(D)** Serum levels of LDH, CK-MB, CK and AST (*n* = 10). The values are presented as the mean ± SEM. ^∗^*P* < 0.05, ^∗∗^*P* < 0.01 vs. DOX, ˆ*P* < 0.05, ˆˆ*P* < 0.01 vs. DOX + GLA, ^@^*P* < 0.01 vs. DOX + Abx.

To determine whether the modulation of gut microbiota dysbiosis by GLA mitigates DOX-induced cardiotoxicity, we used a cocktail of broad-spectrum antibiotics (Abx) to deplete gut microbiota in DOX-treated mice (Figure 3A). The antibacterial efficacy of Abx was confirmed by qPCR (Figure 3B). Not surprisingly, Abx and GLA alone attenuated the effects of DOX on the levels of LPS and butyrate in the feces and peripheral blood and the serum levels of myocardial enzymes including LDH, CK-MB, CK, and AST (Figure 3C,D). The combination of Abx and GLA significantly decreased the levels of butyrate but increased the levels of LPS and myocardial enzyme as compared with the GLA group (Figure 3C,D). Moreover, there was no statistical significance in the levels of myocardial enzymes except AST between the Abx group and GLA plus Abx group (Figure 3D). These data suggest GLA prevents DOX-induced cardiotoxicity through modulation of gut microbiota dysbiosis in mice.

### GLA Switches M1-Like Colonic Macrophages to M2-Like Phenotype in the DOX-Treated Mice

**FIGURE 4:**
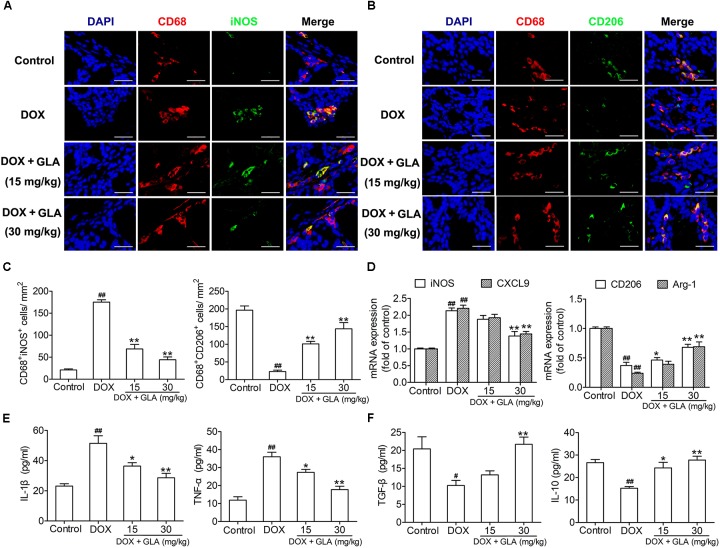
Glabridin (GLA) switches M1-like colonic macrophages to an M2-like phenotype in the doxorubicin (DOX)-treated mice. A single dose of DOX (20 mg/kg) was intraperitoneally injected into the C57BL/6 mice to induce acute cardiotoxicity. GLA (15 and 30 mg/kg) or vehicle was intragastrically administered once daily for 12 days, starting 7 days before DOX injection. **(A,B)** Immunostaining analysis of CD68^+^CD206^+^ or CD68^+^iNOS^+^ macrophages in the colon was shown (*n* = 10). Scale bars, 20 μm. **(C)** Corresponding quantitative analysis of CD68^+^CD206^+^ or CD68^+^iNOS^+^ macrophages (*n* = 10). **(D)** Relative mRNA levels of M1 marker (iNOS and CXCL-9) and M2 markers (CD206 and arginase-1) in the colonic macrophages were measured by qPCR. **(E,F)** M1 cytokines (TNF-α and CCL2) and M2 cytokines (TGF-β and IL-10) in the culture supernatants of colonic macrophages were determined by ELISA (*n* = 10). The values are presented as the mean ± SEM. ^#^*P* < 0.05, ^##^*P* < 0.01 vs. control, ^∗^*P* < 0.05, ^∗∗^*P* < 0.01 vs. DOX.

Cardiotoxicity is aggravated by M1-like macrophage characterized by production of TNF-α and IL-1β, but attenuated by M2-like macrophage characterized by production of TGF-β and IL-10 (Fujiu et al., 2014). Therefore, we determined the colonic macrophage phenotype and their production of cytokines. As shown in Figure 4A–C, DOX significantly increased M1-like (CD68^+^iNOS^+^) cells but decreased M2-like (CD68^+^CD206^+^) cells in the colon when compared with the control, though there was no difference in infiltration of CD68^+^ cells between the two groups. Conversely, GLA dose (15 and 30 mg/kg)-dependently increased M2-like (CD68^+^CD206^+^) cells and decreased M1-like (CD68^+^iNOS^+^) cells in the colon (Figure 4A–C). Similarly, GLA dramatically upregulated M2 genes (arginase-1 and CD206), whereas downregulated M1 genes (iNOS and CXCL9) in the colonic macrophages when compared with the DOX-treated mice (Figure 4D). Moreover, we observed the increased levels of Th2 cytokines (TGF-β and IL-10) and the decreased levels of Th1 cytokines (TNF-α and IL-1β) in the colonic macrophages from the GLA-treated mice when compared with the DOX-treated mice (Figure 4E,F). These results suggest that GLA switches M1-like colonic macrophages to M2-like phenotype in a mice model of DOX-induced cardiotoxicity.

### Effect of Gut Microbiota and Their Products on Cardiomyocyte Apoptosis and Colonic Macrophage Phenotypes in the DOX-Treated Mice

**FIGURE 5:**
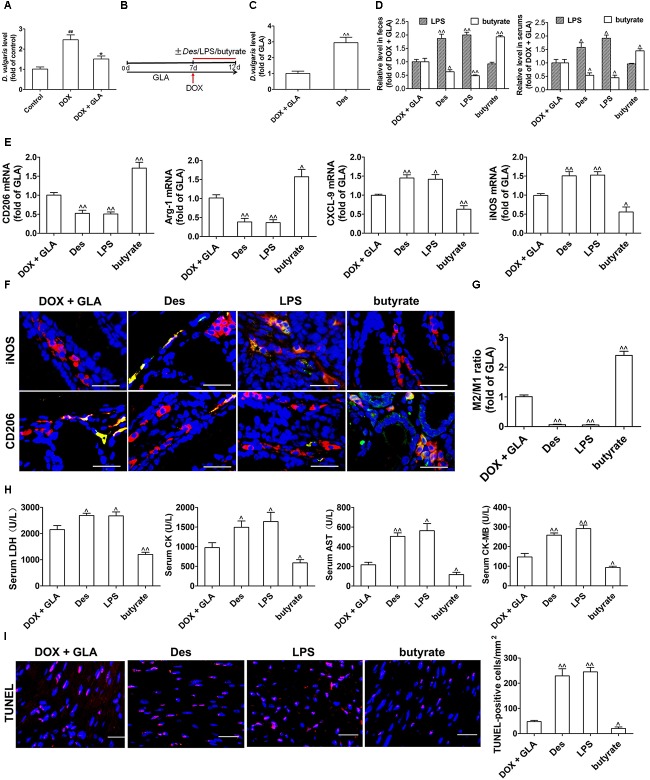
Effect of gut microbiota and their products on cardiomyocyte apoptosis and colonic macrophage phenotypes in the doxorubicin (DOX)-treated mice. A single dose of DOX (20 mg/kg) was intraperitoneally injected into the C57BL/6 mice to induce acute cardiotoxicity. GLA (30 mg/kg) was intragastrically administered once daily for 12 days, starting 7 days before DOX injection. The mice from GLA (30 mg/kg) group were injected intraperitoneally with LPS (1 mg/kg), and orally administrated with sodium butyrate (1 g/kg) or *Desulfovibrio vulgaris* (Des) (1× 10^9^ CFU/mouse/every 2 days) 1 h after the injection of DOX. **(A)** The relative levels of *D. vulgaris* (*n* = 10). **(B)** Schematic representation of the experimental approach. **(C)** The relative levels of *D. vulgaris* (*n* = 10). **(D)** The levels of Lipopolysaccharide (LPS) and butyrate levels in feces and peripheral blood were determined by Limulus Amebocyte Lysate (LAL) assays or high performance liquid chromatography (HPLC), respectively (*n* = 10). **(E)** Relative mRNA levels of M1 marker (iNOS and CXCL-9) and M2 markers (CD206 and arginase-1) in the colonic macrophage were measured by qPCR (*n* = 10). **(F)** Immunostaining analysis of M1-like (CD68^+^CD206^+^) or M1-like (CD68^+^iNOS^+^) macrophages in the colon was shown (*n* = 10). Scale bars, 20 μm. **(G)** Corresponding quantitative analysis of the ratio of M2-like/M1-like macrophages (*n* = 10). **(H)** Serum levels of CK-MB AST, LDH, and CK were assessed (*n* = 10). **(I)** Representative images of the TUNEL assay and quantitative result (*n* = 10). Scale bars, 20 μm. The values are presented as the mean ± SEM. ^#^*P* < 0.05, ^##^*P* < 0.01 vs. control, ^∗^*P* < 0.05, ^∗∗^*P* < 0.01 vs. DOX, ˆ*P* < 0.05, ˆˆ*P* < 0.01 vs. DOX + GLA.

Lipopolysaccharide, mainly from the Gram-negative bacteria in the body, drives DOX-induced cardiotoxicity (Spaan et al., 2008; Wang et al., 2016). Given that *Desulfovibrio* is the only Gram-negative bacteria increased by DOX, we investigated whether *Desulfovibrio* contributed to DOX-induced cardiotoxicity. *D. vulgaris* (Des), the predominant specie of *Desulfovibrio* genus in human and mice colon (Figueiredo et al., 2013; Ritz et al., 2017), was significantly increased by the DOX but decreased by the GLA (Figure 5A). We colonized Des or supplemented its products LPS and butyrate into the DOX plus GLA-treated mice (Figure 5B). The significantly increased colonization of Des was confirmed by qPCR (Figure 5C). As shown in Figure 5D,E, Des increased LPS and decreased butyrate level in the feces and peripheral blood, and increased the expression of M1 markers (iNOS and CXCL9) and decreased the expression of M2 markers (arginase-1 and CD206). Moreover, LPS increased iNOS and CXCL9 levels but butyrate increased arginase-1 and CD206 levels (Figure 5E). We also observed the similar alteration in the proportion of M2-like and M1-like colonic macrophages by immunofluorescence (Figure 5F,G). In addition, myocardial enzyme levels and cardiomyocyte apoptosis were dramatically increased by Des and LPS but decreased by butyrate (Figure 5H,I). We administered butyrate or GLA to the DOX-treated mice and found that they decreased the myocardial enzyme levels (Supplementary Figure S1). These data indicate that GLA reduces DOX-mediated cardiomyocyte apoptosis through gut microbiota modulation and colonic macrophage polarization in mice.

### Modulation of Colonic Macrophage Phenotype by GLA Prevents Cardiomyocyte Apoptosis in the DOX-Treated Mice

**FIGURE 6:**
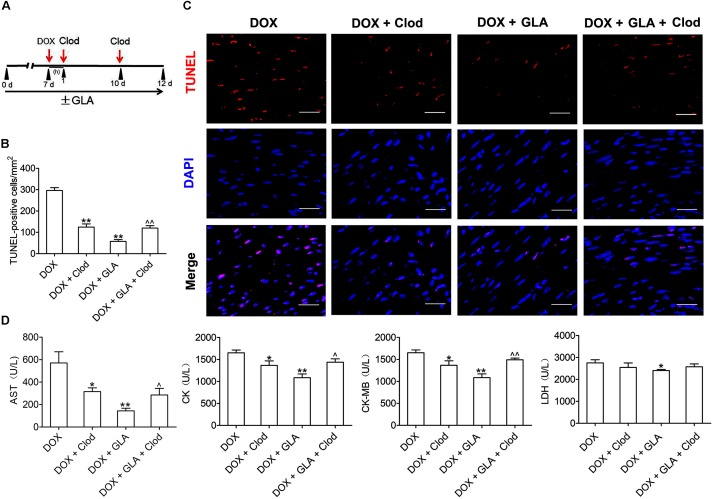
Modulation of colonic macrophage phenotype by Glabridin (GLA) prevents doxorubicin (DOX)-induced cardiomyocyte apoptosis in mice. The C57BL/6 mice from the DOX (20 mg/kg) and GLA (30 mg/kg) groups were administrated intraperitoneally with the clodronate liposome (Clod) (100 mg/kg) 1 h after DOX injection, followed by repeated injections of 50 mg/kg every 4th day for 5 days. **(A)** Schematic representation of the experimental approach. **(B)** Representative images of the TUNEL assay (*n* = 10). Scale bars, 20 μm. **(C)** Quantitative result of TUNEL assay was analyzed (*n* = 10). **(D)** Serum levels of AST, CK, CK-MB, and LDH were assessed (*n* = 10). The values are presented as the mean ± SEM. ^∗^*P* < 0.05, ^∗∗^*P* < 0.01 vs. DOX, ^ˆ^ *P* < 0.05, ^ˆˆ^ *P* < 0.01 vs. DOX + GLA.

To investigate whether the macrophage is responsible for the protective effects of GLA on doxorubicin-induced cardiac injury, we depleted the macrophage by the clodronate liposome (Clod) in the DOX-treated mice, and compared their effects on the DOX-treated mice (containing more M1-polarized colonic macrophages) with the GLA-treated mice (containing more M2-polarized colonic macrophages) (Figure 6A). Elimination of the macrophage decreased the TUNEL positivity of cardiac tissues in the DOX-treated mice, indicating that the colonic macrophage was predominantly of the M1-like pro-inflammation phenotype in these conditions (Figure 6B,C). The TUNEL positivity was reduced by GLA pretreatment, but depletion of the macrophage by Clod increased the TUNEL positivity, suggesting that GLA skews M1-like colonic macrophages to an anti-inflammation M2-like phenotype (Figure 6B,C). Next, we determined the serum levels of AST, CK, CK-MB, and LDH. Unsurprisingly, depletion of the macrophage in the DOX-treated mice decreased the serum levels of AST, CK, CK-MB, and LDH. In contrast, GLA pretreatment reduced the serum levels of the above myocardial enzymes, but macrophage depletion reversed the protective effects of GLA (Figure 6D). These data indicate that GLA prevents DOX-induced cardiomyocyte apoptosis through colonic macrophage phenotype modulation in mice.

### GLA Prevents NF-κB and STAT6 Signaling in the Colonic Macrophage From the DOX-Treated Mice

**FIGURE 7:**
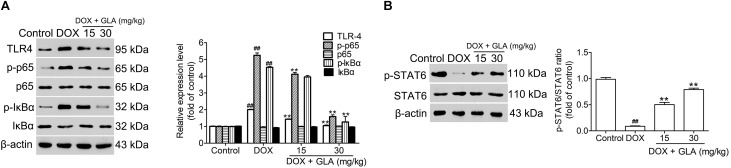
Glabridin (GLA) prevents NF-κB and STAT6 signaling in the colonic macrophage from the doxorubicin (DOX)-treated mice. A single dose of DOX (20 mg/kg) was intraperitoneally injected into the C57BL/6 mice to induce acute cardiotoxicity. GLA (15 and 30 mg/kg) or vehicle was intragastrically administered once daily for 12 days, starting 7 days before DOX injection. **(A)** TLR4, p65, p-p65, p-IκBα, and IκBα in the colonic tissues were determined by Western blot (*n* = 10). **(B)** p-STAT6 and STAT6 in the colonic tissues were determined by Western blot (*n* = 10). The values are presented as the mean ± SEM. ^#^*P* < 0.05, ^##^*P* < 0.01 vs. control, ^∗^*P* < 0.05, ^∗∗^*P* < 0.01 vs. DOX.

NF-κB is a major transcription factor driving the inflammatory gene expression (Schuliga, 2015). We found that DOX treatment increased the levels of TLR4, p-IkBα and p-p65 in the colonic macrophages. In contrast, GLA (15 and 30 mg/kg) decreased the levels of TLR4, p-IkB, and p-p65 without influencing IkBα and p65 (Figure 7A). Butyrate drives M2 macrophage polarization through regulation of STAT6-mediated transcription (Ji et al., 2016). We found that the level of p-STAT6 was decreased by the DOX but increased by the GLA (15 and 30 mg/kg) (Figure 7B). These results suggest that downregulation of NF-κB signaling and upregulation of STAT6 signaling in the colonic macrophages by GLA contributes to the prevention of DOX-induced cardiotoxicity in mice.

## Discussion

Preclinical study shows that flavonoids reduce DOX-induced cardiotoxicity through autoxidation (Shabalala et al., 2017). In the present study, two novel observations have been made. First, we provided direct evidence that flavonoid GLA modulates the DOX-induced dysbiosis of gut microbiota and thereby prevents cardiotoxicity at least partly through *Desulfovibrio* genus. To our knowledge, this is the first study that directly demonstrated the pre-treatment of the GLA inhibited the alteration by the DOX and thereby prevents cardiotoxicity. Second, we demonstrated for the first time that modulation of gut microbiota by the GLA reprograms the colonic macrophages *via* LPS-NF-κB and butyrate-STAT6 signaling. Thus targeting gut microbiota to modulate colonic macrophage response could be a novel strategy for the prevention of the DOX-induced cardiotoxicity. However, our study did not show that *Desulfovibrio* is the sole driver of the increased LPS and decreased butyrate in the DOX-treated mice. Further experiments should be performed to confirm it.

*Desulfovibrio* genus, a Gram-negative LPS-producing bacterium, attributes to the pathogenesis of intestine disorders (Lodowska et al., 2012). Metabolization of SCFAs by *Desulfovibrio* genus exerts a detrimental effect in the patients with colitis (Mills et al., 2008). Removal of gut microbiota was effective in abolishing the damage of the DOX to multiple organs including heart (Wang et al., 2016). Here we demonstrated that treatment with the GLA significantly reduced the abundance of *Desulfovibrio* genus, accompanied by the decreased level of LPS and the increased level of butyrate in feces and peripheral blood in the DOX-treated mice. Though DOX decreased the food intake, previous studies showed that the food restriction for up to 10 weeks does not affect *Desulfovibrio* genus in gut (Henderson et al., 1998; Wang et al., 2018), suggesting that GLA did not prevent DOX-induced cardiotoxicity through affecting food intake. Recent studies show that *Lactobacillus* genus changes the pH of intestinal microenvironment through lactate production, which inhibits growth of *Desulfovibrio* genus (Liévin-Le and Servin, 2014; Zhou et al., 2014). Moreover, *Lactobacillus* in the *Firmicutes* phylum promotes production of butyrate from other gut microbiota, thus exerting beneficial effects in mice with heart failure (Gan et al., 2014; Berni et al., 2017; Liang et al., 2018). These data suggest that flavonoid GLA shows cardioprotective effect in DOX-treated mice through a decrease in *Desulfovibrio* genus and an increase in *Lactobacillus* genus. Helicobacteraceae family promotes inflammatory bowel disease and atherosclerosis (Hansen et al., 2013; Khan et al., 2014). As we unexpected, the abundance of Helicobacteraceae family as Gram-negative LPS-producing bacteria was significantly decreased by DOX but increased by GLA. Further experiments will be carried out to elucidate the underlying mechanism. Previous study showed that treatment of BALB/C mice with DOX at a dose of 10 mg/kg for 2 weeks altered the stool microbiome characterized by the increased *Odoribacter* and decreased *Turicibacter*, *Marvinbryantia*, and *Rikenella* (Jiang et al., 2018). The discrepancy may be due to differences in dose and processing time of DOX and strains of mice. Recent studies showed that pro-inflammatory macrophages significantly decrease gastric bacterial loads especially *Helicobacter pylori* (Viladomiu et al., 2017). Moreover, GLA prevents macrophage M1 polarization in liver and heart (Yehuda et al., 2015). Therefore, further experiments are being carried out to investigate whether GLA changes colonic macrophage polarization, causing change in microbial population.

M1 macrophage significantly aggravates myocarditis, whereas M2 macrophage alleviates myocarditis (Liu et al., 2014; Wang et al., 2017). The progressively increased production of pro-inflammatory cytokines from the cardiac macrophage contributes to the cardiomyopathy (Pecoraro et al., 2016). However, almost nothing has been done on the possible role of the colonic macrophage in DOX-induced cardiotoxicity. Here we demonstrated that DOX treatment upregulated M1 macrophage but downregulated M2 macrophage, which was attenuated by GLA. GLA also decreased production of M1-like cytokines (IL-1β and TNF-α) but increased production of M2-like cytokines (TGF-β and IL-10) in the colonic macrophage. Importantly, macrophage depletion attenuated DOX-induced cardiotoxicity but failed to further affect the effects of GLA. These data suggest that GLA prevents DOX-induced cardiotoxicity though modulation of colonic macrophage phenotype and their production of multiple cytokines. Thus, targeting the colonic macrophage not any single cytokine may represent a novel strategy for the prevention of DOX-induced cardiotoxicity.

Soybean isoflavones ameliorate ischemic cardiomyopathy by activating antioxidant responses (Li and Zhang, 2017). GLA prevents LDL-induced cardiovascular injury and atherogenic processes (Somjen et al., 2004; Kang et al., 2015). Here we demonstrated that GLA (15 and 30 mg/kg) ameliorated DOX-induced cardiotoxicity in mice, clearly revealed by a decrease in serum levels of myocardial enzyme and staining of TUNEL positive nuclear and Masson’s Trichrome. Importantly, GLA downregulated pro-apoptotic proteins (Bax, cleaved-caspase 9 and cleaved-caspase 3) and upregulated anti-apoptotic proteins (HAX-1 and Bcl-2). Previous study showed that treatment with GLA (160 mg/kg/d) for consecutively 4 weeks does not provide any evidence of chronic toxicity (Lee et al., 2012). These data suggest that GLA prevents DOX-induced cardiotoxicity with little side-effect. The concentration of GLA in plasma is found very low in 5 and 20 mg/kg-treated mice, and only about 7.5% of GLA is absorbed (Cao et al., 2007). Moreover, GLA attenuates colonic inflammation in mice induced by dextran sulfate sodium, and exhibits spectrum antibiotic activities against pathogen in colon (Kwon et al., 2008; Simmler et al., 2013), suggesting most of GLA passes to the colon and changes gut microbiota. Gut microbiota can directly affect drug response through influencing their bioavailability or bioactivity (Spanogiannopoulos et al., 2016). Recent study showed that Abx inhibit the clinical benefit of PD-1 blockers in cancer patients (Routy et al., 2018). Similarly, we found that Abx and GLA alone prevented DOX-induced cardiotoxicity, but the combination of GLA with Abx did not decrease the DOX-induced cardiotoxicity. Moreover, human gut microbiota transforms or degrades DOX only in the intestinal tract (Yan et al., 2018), suggesting they could prevent DOX-induced damage to gut. Although it is possible that GLA alters the ability of the gut microbiota to transform DOX in gut, it could not prevent DOX-induced cardiotoxicity.

**FIGURE 8:**
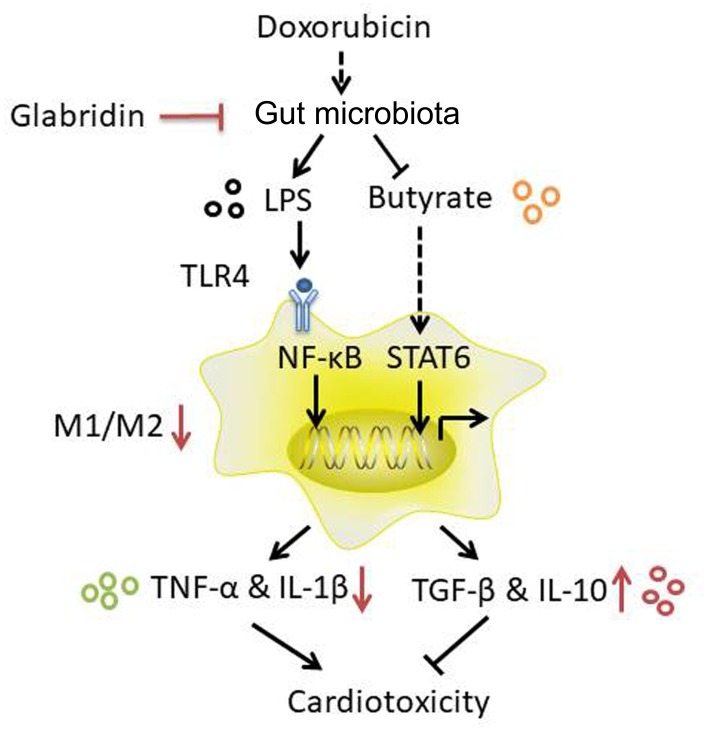
A proposed mechanism to explain the role of the glabridin (GLA) in doxorubicin (DOX)-induced cardiotoxicity.

Taken together, modulation of gut microbiota by GLA prevents DOX-induced cardiotoxicity in mice through decreasing the proportion of M1/M2-like colonic macrophages *via* LPS-NF-κB and butyrate-STAT6 signaling (Figure 8). Importantly, our study identifies previously unknown role of gut microbiota-mediated alteration of colonic macrophage phenotype in DOX-induced cardiotoxicity. Our findings suggest that flavonoid GLA alleviates DOX-induced apoptosis of the myocardial cell through gut microbiota modulation and colonic macrophage polarization, and may provide a potential preventive strategy for DOX-induced cardiotoxicity.

## Author Contributions

KH and XZ conceived and designed the experiments. KH performed most of the experiments and contributed to the manuscript preparation. HT and MQ proposed and helped in editing this article. YL, CL, CD, CW, and JY edited the article. All authors read and approved the final manuscript.

## Conflict of Interest Statement

The authors declare that the research was conducted in the absence of any commercial or financial relationships that could be construed as a potential conflict of interest.
