# Supplementary material for: Glabridin Prevents Doxorubicin-Induced Cardiotoxicity Through Gut Microbiota Modulation and Colonic Macrophage Polarization in Mice
Source: Front Pharmacol. 2019 Feb 15;10:107. doi: 10.3389/fphar.2019.00107 (PMC6387923; doi:10.3389/fphar.2019.00107)
Supplement: Supplementary file 1 [file Data_Sheet_1.docx]

**Glabridin prevents doxorubicin-induced cardiotoxicity through gut microbiota modulation and colonic macrophage polarization in mice**

Keqing Huang^1^, Yanzhuo Liu^2,3^, Honglin Tang^2^, Miao Qiu^2,4^, Chenhong Li^5^, Chenfan Duan^2^, Chenlong Wang^2^, Jing Yang^2^, Xiaoyang Zhou^1,*^

**Supplementary table 1**

Primers used for real-time PCR to detect the mRNAs of target genes.

| Primers | Forward primer (5’-3’) | Reverse primer (5’-3’) |
| --- | --- | --- |
| iNOS | GTTCTCAGCCCAACAATACAAGA | GTGGACGGGTCGATGTCAC |
| CXCL9 | CCGAGGCACGTCCACTTACA | TCTAGGTTTGATCCCGTTC |
| CD206 | CAAGGAAGGTTGGCATTTGT | CCTTTCAGTCCTTTGCAAG |
| arginase-1 | CTCCAAGCCAAAGTCCTTAGAG | AGGAGCTGTCATTAGGGACATC |
| β-actin | TGACAGGATGCAGAAGGAGA | TAGAGCCACCAATCCACACA |
| *Desulfovibrio vulgaris* | CCTAGGGCTACACACGTACTACAA | GAGCATGCTGATCTCGAATTACTA |

**Supplementary picture**


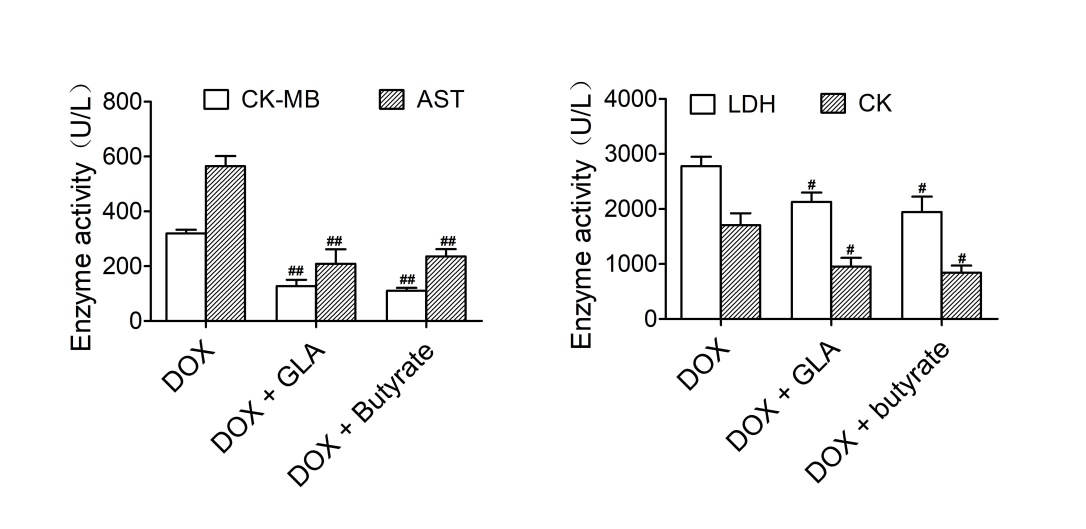


Fig. S1. Effect of gut microbiota and their products on cardiomyocyte apoptosis and colonic macrophage phenotypes in the doxorubicin (DOX)-treated mice. A single dose of DOX (20 mg/kg) was intraperitoneally injected into the C57BL/6 mice to induce acute cardiotoxicity. GLA (30 mg/kg) was intragastrically administered once daily for 12 days, starting 7 days before DOX injection. Sodium butyrate (1 g/kg) was intragastrically administered once daily for 7 days 1 h after the injection of DOX. Serum levels of CK-MB AST, LDH, and CK were assessed (n = 10). The values are presented as the mean ± SEM. ^*^*P* < 0.05, ^**^*P* < 0.01 vs. DOX.
